# DISCRIMINATION, STRESS, AND MORTALITY AMONG BLACKS AND WHITES

**DOI:** 10.1093/geroni/igz038.2653

**Published:** 2019-11-08

**Authors:** Soyoung Choun, Sungrok Kang, Hyunyup Lee, Carolyn M Aldwin

**Affiliations:** 1 Oregon State University, Corvallis, Oregon, United States; 2 Korea Military Academy, Seoul, Korea, Republic of

## Abstract

Mortality rates have declined significantly in the past decades. However, Case and Deaton (2015) showed that middle-aged white Americans with lower education levels have increasing mortality rates. Although some have suggested that stress is an important factor in both this and in racial/ethnic disparities in mortality, relatively few studies have examined vulnerability to stress and mortality, and typically have examined only one type of stress. We examined racial/ethnic and gender differences in different types of stressors, from everyday discrimination, to lifetime trauma, as well as differential mortality risk due to stress vulnerability. Using data from the Health and Retirement Study (HRS), the sample consisted of 6,810 (Mage=68.9 years, SD=10.1) who completed the Psychosocial Questionnaire (PQ) in 2006; mortality was assessed to 2014. Blacks were higher on most stressors except for lifetime trauma. Women reported higher level of financial strain but lower levels of everyday discrimination and lifetime trauma than men. Controlling for demographics and self-rated health, Cox proportional hazard models revealed that everyday discrimination, financial strain, SLEs, lifetime trauma were significantly associated with the risk of mortality. There were no significant racial/ethnic differences in mortality risk. However, interaction effects showed that whites had higher mortality risk with lifetime trauma than Blacks, while those with lower education had higher mortality risk for SLEs. This supports the idea that lower education whites may be more susceptible to some types of stressors, providing a possible mechanism for Case and Deaton’s finding (2015) of increasing mortality risk in this group.

